# Recent advances: fertility preservation and fertility restoration options for males and females

**DOI:** 10.12703/r/10-55

**Published:** 2021-06-10

**Authors:** Chatchanan Doungkamchan, Kyle E Orwig

**Affiliations:** 1 Molecular Genetics and Developmental Biology Graduate Program, University of Pittsburgh School of Medicine, Pittsburgh, PA 15260, USA; 2 Department of Obstetrics, Gynecology and Reproductive Sciences, Magee-Womens Research Institute, University of Pittsburgh School of Medicine, Pittsburgh, PA 15213, USA; 3 Department of Anatomy, Faculty of Medicine, Chulalongkorn University, Bangkok, Thailand

**Keywords:** Fertility preservation, male fertility preservation, female fertility preservation, cryopreservation, transplantation, ovarian tissue cryopreservation, testicular tissue cryopreservation, stem cells

## Abstract

Fertility preservation is the process of saving gametes, embryos, gonadal tissues and/or gonadal cells for individuals who are at risk of infertility due to disease, medical treatments, age, genetics, or other circumstances. Adult patients have the options to preserve eggs, sperm, or embryos that can be used in the future to produce biologically related offspring with assisted reproductive technologies. These options are not available to all adults or to children who are not yet producing mature eggs or sperm. Gonadal cells/tissues have been frozen for several thousands of those patients worldwide with anticipation that new reproductive technologies will be available in the future. Therefore, the fertility preservation medical and research communities are obligated to responsibly develop next-generation reproductive technologies and translate them into clinical practice. We briefly describe standard options to preserve and restore fertility, but the emphasis of this review is on experimental options, including an assessment of readiness for translation to the human fertility clinic.

## Introduction

Fertility preservation is a process to protect or preserve the ability of individuals to have their own biological children by freezing sperm, eggs, embryos, or gonadal tissues/cells^1^. Fertility preservation is indicated in patients with reproductive potential who are at risk of infertility (for example, azoospermia or premature ovarian insufficiency) or reduced fertility (oligospermia or diminished ovarian reserve) due to their disease, gonadotoxic medical treatments, age, or other circumstances^2–6^. Patients at risk of infertility should be counseled about their risk and referred to reproductive specialists to discuss options for fertility preservation, which include both standard-of-care and experimental approaches^2,5–11^. We will briefly list the established standard-of-care fertility preservation options and describe in more detail experimental options to preserve and restore the fertility of women, men, girls, and boys who are at risk of infertility.

## Male

**Figure 1.**
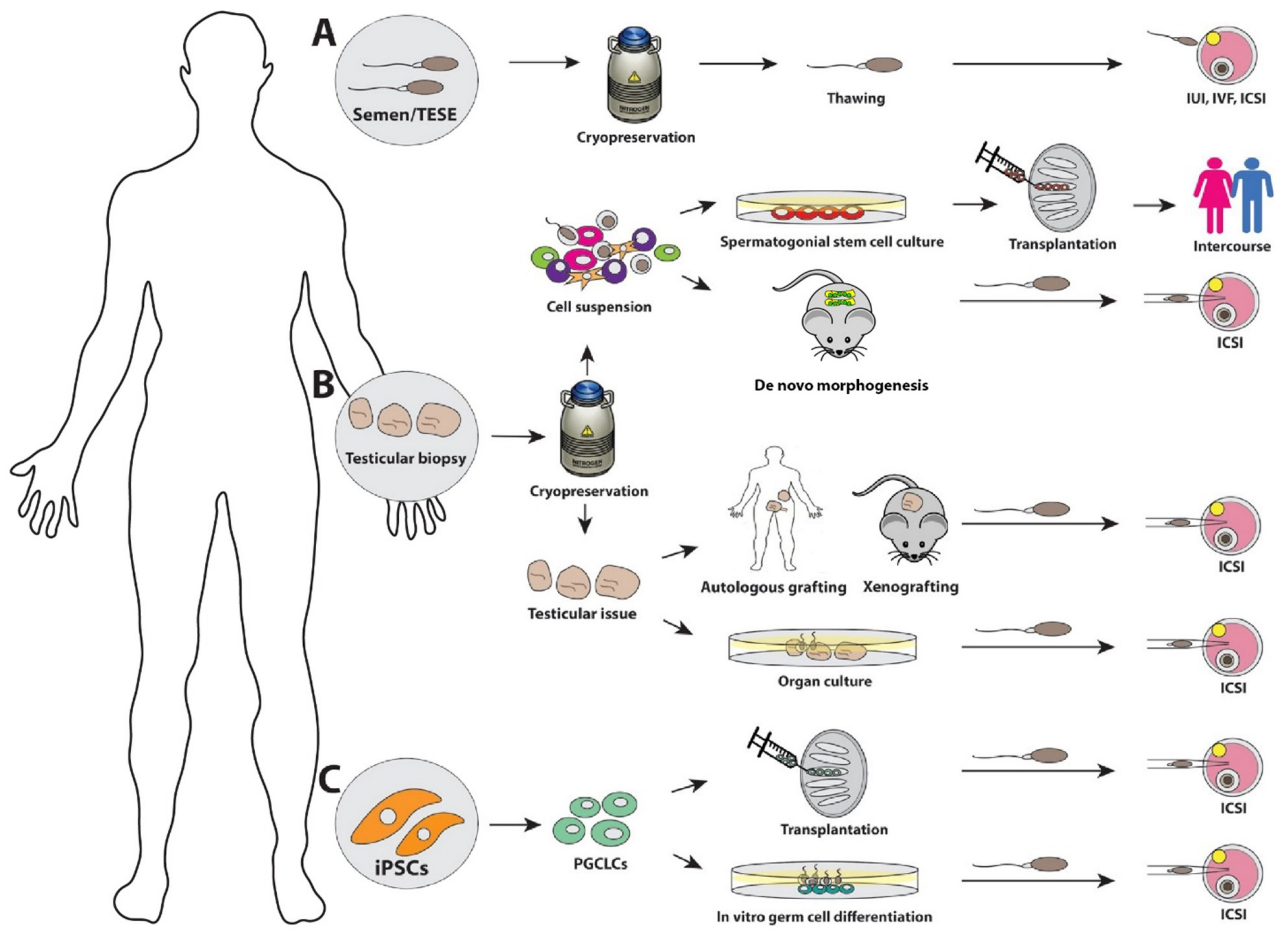
Fertility preservation methods for male patients. Sperm cryopreservation (**A**) is a standard-of-care practice to preserve fertility in adult male patients. Although this option is not feasible in patients who do not have mature sperm, testicular tissue can be cryopreserved under experimental protocol (**B**). The downstream applications for cryopreserved testicular tissue include spermatogonial stem cell transplantation with or without spermatogonial stem cell culture, *de novo* testicular morphogenesis, testicular tissue grafting/xenografting, and testicular tissue organ culture. *In vitro* germ cell technologies in which germ cells or gametes are produced from patient somatic cells are in the early stages of development. Induced pluripotent stem cells (iPSCs), which are derived from a patient’s somatic cells (for example, skin fibroblasts), can be differentiated into primordial germ cell-like cells (PGCLCs), which can be transplanted into the seminiferous tubules to make sperm or differentiated to sperm *in vitro* (**C**). ICSI, intracyctoplasmic sperm injection; IUI, intrauterine insemination; IVF, *in vitro* fertilization; TESE, testicular extraction of sperm.

Sperm cryopreservation is the established standard-of-care approach to preserve reproductive potential in adolescent and adult male patients (Figure 1A)^2,4–6^. Sperm cryopreservation is not possible for prepubertal patients who are not producing sperm or transgender females receiving medical treatments to suppress testosterone. The only option to preserve the reproductive potential of those patients who are not producing sperm is testicular tissue cryopreservation, which is investigational and should be done under approved protocols (Figure 1B)^2,4,5,12^. In this section, we will focus on the investigational cell- and tissue-based methods to produce sperm or restore fertility in males who have cryopreserved testicular tissues (Figure 1B). The experimental method in which sperm are derived from somatic cells through induced pluripotent stem cells (iPSCs) and primordial germ cell-like cells (PGCLCs) (Figure 1C) will be covered separately at the end of this review.

## Cell-based therapies

### Spermatogonial stem cell transplantation

Cryopreserved immature testicular tissues contain spermatogonial stem cells (SSCs) with the potential to initiate spermatogenesis, the process that produces sperm. Those tissues can be thawed and digested with enzymes to produce a testicular cell suspension, which contains SSCs, and subsequently transplanted into the seminiferous tubules of the testes to regenerate spermatogenesis and potentially restore fertility. This technique was first described in mice by Brinster *et al*.^13,14^ and has since been translated to rats, dogs, goats, sheep, pigs, and monkeys. The studies reported sperm in dogs, cattle, pigs, and monkeys^15–18^; hatching blastocysts in monkeys^19^; and offspring in mice, rats, goats, and sheep^20–27^. Therefore, it may be possible that immature testicular tissues from prepubertal male patients can be cryopreserved prior to gonadotoxic medical treatments, thawed at a later date, and digested with enzymes to produce a testicular cell suspension that is transplanted to regenerate spermatogenesis and possibly restore fertility. Autologous transplantation of frozen and thawed testis cells was reported in seven adult survivors of Hodgkin’s lymphoma in 2003, but reproductive outcomes of that study have not been reported^28^. Testicular tissue cryopreservation has now been reported for patients worldwide^29–38^ and some of those patients may be ready to use their tissues to have biological children. SSC transplantation is a mature technology that may be ready for translation to the human clinic. All approaches to use cryopreserved testicular tissues for reproduction are experimental and should be conducted with ethical approval and full reporting of safety and reproductive outcomes.

The tissue biopsies from young patients are usually small and may not contain a sufficient number of SSCs to produce robust spermatogenesis after transplantation. Thus, it may be necessary to expand SSC numbers in culture before transplantation. SSC culture has been firmly established in rodents^39–44^, including development of conditions that do not require supporting feeder cells^45,46^, which may be an important consideration for clinical application. SSC culture has been extended to rats, hamsters, and rabbits^41,47,48^, but extension to larger animal species has been a challenge and this is perhaps due to species-specific differences in SSC regulation^49–51^. Many laboratories have described protocols for human SSC culture^32,52–68^, but definitive evidence of long-term SSC expansion in higher primates is lacking, and no methods have been independently replicated among laboratories^61,69,70^. The slow cell cycle of SSCs in higher primates may partially explain the difficulty expanding those cells in culture^71^.

### *De novo* testicular morphogenesis

*De novo* testicular morphogenesis is another cell-based therapy to regenerate spermatogenesis from testicular cell suspensions. Testicular cell suspensions consist of germ cells and somatic cells such as Sertoli cells, Leydig cells, peritubular myoid cells, endothelial cells, immune cells, and fibroblasts. Testicular cell suspensions from neonatal or fetal animals can reorganize to form seminiferous tubule-like structures when put under the skin of immune-deficient mice (*de novo* testicular morphogenesis)^72–75^. *De novo* morphogenesis has been reported to produce elongated spermatids in pig and sheep^74,75^ and round spermatids that resulted in live-born offspring in mice^73^. There have been no reports of *de novo* testicular morphogenesis from human testis cells to date.

## Tissue-based therapies

### Autologous testicular tissue grafting

Testicular tissue grafting is an alternative to SSC transplantation. In this approach, SSCs are maintained in their cognate seminiferous tubule niches in intact pieces of testicular tissue. Homologous species testicular tissue grafting was pioneered in mice, demonstrating that immature mouse testicular tissues could be grafted under the back skin of recipient mice and matured to produce complete spermatogenesis^76–78^. Graft-derived sperm were competent to fertilize mouse eggs and produce healthy mouse offspring^77,78^.

Four studies have reported autologous and/or homologous grafting of immature nonhuman primate testicular tissues^79–82^, including studies that demonstrated the production of sperm^80–82^ and a healthy baby^82^ from cryopreserved tissues. Therefore, autologous grafting of cryopreserved prepubertal testicular tissues is a mature technology that may be ready for translation to the human clinic.

### Testicular tissue xenografting

Autologous testicular tissue grafting may not be appropriate for patients who harbor malignant cells in their testicular tissues (for example, leukemia or testicular cancer) or transgender females who do not want to experience male puberty, including testosterone production that is required to mature testicular tissues and produce sperm. Xenografting of immature testicular tissues to an animal host may provide an alternative to produce sperm outside the patient’s body. Immature testicular tissues from pigs, goats, rabbits, hamsters, dogs, cats, horses, cattle, and monkeys have been grafted under the back skin of immune-deficient nude mice and matured to produce spermatids or sperm (reviewed in 83). Xenograft-derived sperm recovered from mouse hosts have been used to fertilize and produce embryos or offspring in rabbits, pigs, and monkeys^77,84–86^. Xenografting of immature human testicular tissues has failed to produce sperm to date^87–92^ and this approach may raise concerns about transmission of xenobiotics if used in the clinic. However, if proven effective, testicular tissue xenografting may be an approach to circumvent cancer contamination problems or for transgender individuals who will not go through male puberty and cannot mature testicular tissues inside their own bodies.

### Testicular organ culture

In 2011, Sato *et al*. demonstrated in mice that fresh or cryopreserved neonatal testicular tissues could be matured in organ culture on an island of agar at the air–liquid interface to produce mature sperm^93^. Sperm from fresh tissues were tested functionally by fertilization and with the production of offspring^93^. The same group later showed that cryopreserved testicular tissues could be matured to produce sperm and offspring; this has important implications for adult survivors of childhood cancers who have cryopreserved their testicular tissues^94^. This approach to mature testicular tissues outside the body also has important implications for transgender females who are not producing testosterone and will not be able to mature testicular tissues inside their bodies. Long-term tissue survival was not observed using the initial agar-island culture system, so Komeya *et al*. developed a pump-driven microfluidic device that maintained tissues for at least 6 months with production of sperm and offspring from fresh tissues^95^. The same group later introduced a simpler pumpless microfluidic device that eliminated the dependence on pumps and tubes and supported testicular tissue maturation and sperm production for at least 4 months^96^. Offspring were not produced from cryopreserved tissues using either the pump-driven or pumpless device. These results have not been replicated in any higher animal models where sperm function could be tested by fertilization and production of offspring. Human testicular tissues have been cultured with results ranging from tissue survival with spermatogonia^97–99^ or spermatocytes^100^ or round spermatids^101^ as the most advanced stage. Functional validation of haploid germ cells from human tissues is difficult or impossible because of ethical, legal and/or funding restrictions. Therefore, studies in large animal models are necessary to demonstrate feasibility and safety before translation to the clinic should be considered.

## Female

**Figure 2.**
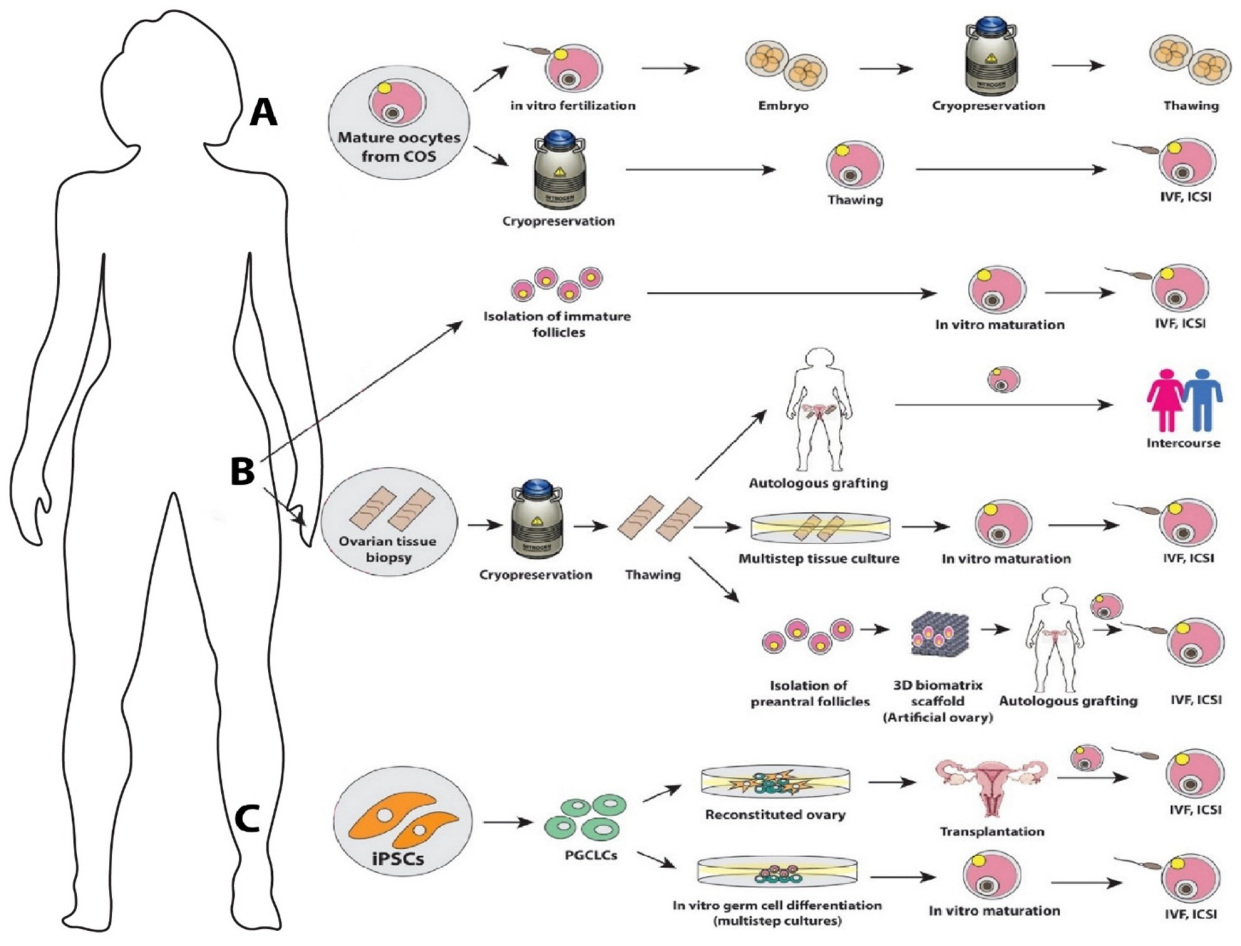
Fertility preservation methods in female patients. Standard practice recommended by the American Society of Clinical Oncology and the American Society for Reproductive Medicine for female patients with cancer is to cryopreserve embryos or mature oocytes in some cases (**A**). Ovarian tissue cryopreservation or immature oocyte cryopreservation (or both) for *in vitro* maturation are investigational methods for prepubertal girls or patients whose cancer treatment cannot be delayed (**B**). Downstream utilization of cryopreserved ovarian tissues includes autologous tissue grafting, ovarian tissue culture for *in vitro* growth and *in vitro* maturation of primordial/primary follicles, and artificial ovary implantation. Derivation of oocytes from induced pluripotent stem cells (iPSCs) is in the early stages of development (**C**). COS, controlled ovarian stimulation; ICSI, intracyctoplasmic sperm injection; IVF, *in vitro* fertilization; PGCLC, primordial germ cell-like cell.

Standard-of-care fertility preservation options for women include (1) embryo cryopreservation, (2) mature oocyte cryopreservation, and (3) ovarian shielding or transposition in patients undergoing radiotherapy (Figure 2A)^2,12^. Ovarian tissue cryopreservation is an option for prepubertal girls who are not producing mature oocytes or women who cannot undergo controlled ovarian stimulation for oocyte collection because of time constraints or other concerns (Figure 2B)^2,3,5,9,102^. The American Society of Reproductive Medicine has advised that the experimental label can be removed from ovarian tissue cryopreservation^12^ on the basis of the growing number of live offspring that have been reported after transplantation of ovarian tissue^3^. Other investigational methods, including *in vitro* maturation (IVM) of immature oocytes, *in vitro* growth of primordial/primary follicles, non-antral/small antral follicles, and artificial ovary, will also be reviewed in the following sections (Figure 1B). The experimental methods regarding *in vitro* gametes from pluripotent stem cells (Figure 2C) will be reviewed in the last section (‘*In vitro* gametes from pluripotent stem cells’).

### Ovarian tissue cryopreservation and autologous tissue grafting

Ovarian tissue cryopreservation is the only option for prepubertal female patients with cancer or for adult women with estrogen-sensitive cancer or those whose cancer treatment cannot be delayed^2,3,5,9,102^. Cryopreserved ovarian tissues can be reimplanted to the patients, either in the pelvic cavity or on the ovarian medulla (orthotopic transplantation) or outside the peritoneal cavity (heterotopic transplantation), if the risk of transferring cancer is low^9,102,103^. Orthotopic reimplantation of cryopreserved ovarian tissues performed in human patients restored ovarian function in more than 90% of cases^104,105^. The duration of ovarian activity ranged from 4 to 7 years after reimplantation^104,106^. The pregnancy rate is 18 to 35% of transplanted cases, and the live birth rate is 13.6 to 25% of transplanted cases^107–109^. The reimplantation outcome may be improved by enhancing graft revascularization by angiogenic and antiapoptotic factors^3^. Ovarian tissue transplantation is available at only a few centers worldwide. The hormonal and live birth outcomes from transplanted ovarian tissues are encouraging and may justify wider implementation in centers with the knowledge, expertise, and infrastructure to conduct the procedure safely and with the appropriate regulatory approval.

### *In vitro* maturation of immature follicles

Although the ovarian tissue cryopreservation coupled with transplantation is successful in humans, it may not be appropriate in cases where there is a risk of malignant contamination^110,111^, such as patients with ovarian cancers, blood-borne malignancies, cancers with potential to metastasize to the ovaries, or a genetic predisposition to ovarian cancer^9,112^. In addition, ovarian tissue transplantation may not be appropriate for transgender males because of estrogen production associated with follicle development. IVM may allow maturing of unstimulated or minimally stimulated oocytes from antral/germinal vesicle follicles to fertilization-competent metaphase II oocytes *in vitro*^11,113,114^, hence decreasing the chance of reintroducing cancer^115^. Additionally, immature follicles can be collected with minimal or no ovarian stimulation which is beneficial in the cancer patients whose cancer is hormone-sensitive, patients who cannot delay chemotherapy, prepubertal patients who are not sexually mature, and patients with polycystic ovarian syndrome (PCOS) to avoid ovarian hyperstimulation syndrome^11,116^. Immature follicles can be harvested directly by aspiration from the ovary, *in situ* or after oophorectomy in patients with ovarian cancer, or during processing of ovarian cortex for ovarian tissue freezing^11,117–120^.

Obstetrics outcomes of IVM were reported among different disease status patients, such as PCOS or PCO-like patients, patients with cancer, or infertile women with no underlying diseases, regardless of whether they had been treated with a hormonal priming regimen prior to oocyte retrieval (reviewed in 11,114,116,121). Studies from six centers reported about 400 live births with no increase in birth anomalies when compared with conventional *in vitro* fertilization (IVF)^122–128^.

### *In vitro* growth of primordial follicles by multistep culture technique

About 90% of the follicles in ovarian tissue biopsies are primordial follicles, which cannot survive or be matured when cultured under regular IVM conditions^129^. To address this problem, a dynamic multistep culture technique for *in vitro* growth of oocytes was developed for growing primordial follicles to the stage that can be matured by IVM technique. Dynamic multistep culture mimics follicular development *in vivo* and is generally composed of three steps: (1) activation of primordial follicles to small preantral follicles, (2) *in vitro* growth of small preantral follicles to antral follicles where cumulus–oocyte complexes (COCs) can be isolated for (3) IVM^130–132^.

*In vitro* growth of primordial follicles was shown in mice to produce liveborn offspring^133,134^. In humans, culturing oocytes *in situ* with the ovarian tissue was shown to support primordial follicle activation followed by *in vitro* growth of small preantral follicles to antral follicles in which COCs can be used for IVM, resulting in metaphase II mature oocyte all *in vitro*^132,135,136^. These multistep culture techniques therefore may broaden the utility of cryopreserved ovarian tissue.

### Artificial ovary implantation

An alternative approach for the cases with a higher risk of transferring cancer is to extract primordial follicles from cryopreserved ovarian tissues, put them on a supporting scaffold, and graft back to the patients or immunocompromised mice. These techniques have been shown to produce offspring in mice and supported short-term growth of preantral follicles after xenografting of human primordial follicles into severe combined immunodeficient (SCID) mice^137–142^. This technique is another way of using cryopreserved ovarian tissue without reintroducing the whole tissue and potentially cancerous cells back to the patients.

## *In vitro* gametes from pluripotent stem cells

Counseling, consenting, and freezing of gonadal tissues for fertility preservation should be carried out before treatment or early in treatment before the oogenic or spermatogenic potential of the ovaries or testes is permanently destroyed. Parenthood is important to cancer survivors^143–145^. However, despite the best intentions of patients, families, and medical professionals, fertility preservation can be difficult to accomplish in the compressed and stressful time frame between diagnosis and initiation of treatment^30,37,38,105,146–149^.

In the future, it may not be necessary to cryopreserve gonadal tissues before treatment because it will be possible to produce eggs, sperm, or their precursors from skin cells or other somatic cells of the body. In this short review, it is impossible to detail all of the outstanding studies that laid the foundation for the emerging discipline of “*in vitro* germ cells” or “*in vitro* gametogenesis”, but references are provided here. This field emerged from early observations that germ cells sometimes spontaneously arise from pluripotent embryonic stem cells (ESCs) or iPSCs in two-dimensional or three-dimensional cultures after removal of leukemia inhibitory factor (LIF) from the culture medium^150–161^. Those events were rare and stochastic. Studies on Bmp4-deficient embryos and cultures of mouse epiblasts or epiblast stem cells provided initial clues about early germ cell markers as well as growth factors, signaling pathways, and transcription factors that were important for germ cell specification^162–169^. That knowledge was subsequently exploited to induce germ cell differentiation from pluripotent cells, as described above.

Hayashi *et al*. provided the proof of principle for this approach in mice by demonstrating that ESCs or iPSCs could be differentiated into epiblast-like cells and then to PGCLCs that were transplanted to the ovaries or testes to produce eggs, sperm, and live offspring^170–172^. In subsequent studies, PGCLCs were differentiated to haploid eggs or spermatids entirely *in vitro*. The resulting eggs and sperm were fertilization-competent and produced offspring^173,174^. Germ cells have also been produced from monkey and human pluripotent stem cells^169,175–179^. Monkey and human PGCLCs are transplantable (produce clusters of spermatogonia or oogonia) but so far there is no evidence that they can differentiate to produce fertilization-competent gametes, *in vivo* or *in vitro*^177,178,180–182^. This may be more a reflection of experimental limitations than actual limitations of PGCLC developmental potential in higher primates.

## Concluding remarks

Assisted reproductive technologies enable fertility preservation for adolescent or adult patients who are able to produce eggs or sperm. Gonadal tissue cryopreservation is an investigational option to preserve the reproductive potential of patients who cannot produce eggs or sperm. Gonadal tissue freezing has been performed for thousands of patients worldwide, demonstrating that the methods to obtain tissues are safe and feasible^29,30,183,184^. However, access to gonadal tissue freezing is limited primarily to academic centers and is performed under institutionally approved experimental protocols. A few centers are addressing the geographical access-to-care challenge by providing centralized tissue processing and freezing services. This allows patients to have surgery performed at their local institutions and have tissue express-shipped to a center with the infrastructure, expertise, and capacity to process, freeze, and store tissues^30,185,186^. Access to care is also limited because costs for these experimental fertility preservation options are typically paid out of pocket. The experimental designation is because there is limited evidence that cryopreserved immature gonadal tissues can be used for reproduction.

This review describes several methods in the research pipeline to mature gonadal tissues by transplantation or by IVM to produce fertilization-competent eggs or sperm. Ovarian tissue transplantation was introduced to the clinic in 2004 and has led to over 130 live births to date^3,187,188^. Those live birth outcomes prompted the American Society for Reproductive Medicine to recommend that the experimental label could be removed from ovarian tissue cryopreservation^12^, which may open the door to insurance coverage in some locations. Notably, however, it was not always possible to unequivocally document that pregnancies resulted from the transplanted tissue versus endogenous cells, and there is only one published report of a birth from ovarian tissue that was frozen during childhood^189^.

SSC transplantation and testicular tissue grafting for males are mature technologies that may be ready for translation to the human clinic^19,82^. There are no human live births from frozen/thawed testicular tissues, so the fertility restoration option remains experimental in the United States. Testicular tissues, in contrast to ovarian tissues that are cryopreserved for adult women and prepubertal girls, are cryopreserved almost exclusively for prepubertal boys. That means it could be many years before the first males return to use their cryopreserved testicular tissues. How many more years and how many births will be required to remove the experimental label from testicular tissue freezing? If a man produces sperm or offspring (or both) after autologous transplantation of SSCs, how will we know whether sperm were from transplanted or endogenous cells? The Danish experience may be instructive. In 1998, the Danish Minister of Health concluded that there were no restrictions on freezing ovarian tissues or testicular tissues as long as only autologous transplantation was considered. This liberal ruling placed gonadal tissue freezing in the context of normal medical practice^183^.

*In vitro* germ cells or *in vitro* gametogenesis are emerging technologies that could eliminate the need to cryopreserve gonadal tissues prior to treatment. Pioneering results in mice are promising, but it is important to acknowledge that there has been limited independent replication of the original fertile outcomes^170–174^ in mice and, to our knowledge, no replication in higher animal models. Several labs have reported the production of transplantable PGCLCs from monkey or human pluripotent stem cells, but there is no evidence to date that those cells can produce functional gametes, *in vivo* or *in vitro*^177,180–182,190^. The gold standard to prove the gametogenic potential of PGCLCs is to demonstrate that PGCLC-derived gametes can fertilize and produce healthy offspring. Those endpoints are nearly inaccessible under current regulatory and funding restrictions for human embryo research in the US. Producing human embryos for research is illegal in our state (Pennsylvania). Circumstances in other states or countries may be more permissive^191^. Nonhuman primate studies are a good preclinical surrogate to establish safety and feasibility but are expensive and accessible to only a few researchers. Nonetheless, more basic and translational research, including research in higher primates, is needed to demonstrate the safety, feasibility, and reproducibility of *in vitro* germ cell technologies.

*In vitro* germ cell technologies may also bring a higher regulatory burden. The US Food and Drug Administration guidance for transplantation of human cells, tissues, and cellular and tissue-based products (HCT/Ps) stipulates that HCT/Ps that meet specific criteria or fall within detailed exceptions do not require premarket review and approval. To qualify for exception, the HCT/Ps must be minimally manipulated, be intended for homologous use (same function), and be for autologous or reproductive use or both. The *in vitro* reprogramming and stepwise differentiation required to convert a somatic cell to a germ cell or gamete may exceed the definition of homologous use and minimal manipulation, thus requiring regulation as a drug, device and/or biological product.

It is an exciting time in reproductive biology and reproductive medicine. The technology that produced the word’s first IVF baby, Louise Brown (born July 25, 1978), has now produced millions of babies worldwide. Louise was possible because her mother could produce eggs and her father could produce sperm that were combined in the IVF laboratory of Drs. Steptoe (surgeon) and Edwards (researcher) at Oldham General Hospital in the UK^192^. This review describes a next generation of reproductive technologies that may enable patients with the most difficult infertility diagnoses (no eggs, no sperm) to have biologically related children. Development of these technologies, like that of IVF, will require the coordinated efforts of researchers and physicians and must be deployed in a transparent manner with regulatory oversight and with input from key stakeholders.

## Abbreviations

COC, cumulus–oocyte complex; ESC, embryonic stem cell; HCT/P, human cells, tissues, and cellular and tissue-based products; iPSC, induced pluripotent stem cell; IVF, *in vitro* fertilization; IVM, *in vitro* maturation; PCOS, polycystic ovarian syndrome; PGCLC, primordial germ cell-like cell; SSC, spermatogonial stem cell
